# Association of the Host Immune Response with Protection Using a Live Attenuated African Swine Fever Virus Model

**DOI:** 10.3390/v8100291

**Published:** 2016-10-22

**Authors:** Jolene Carlson, Vivian O’Donnell, Marialexia Alfano, Lauro Velazquez Salinas, Lauren G. Holinka, Peter W. Krug, Douglas P. Gladue, Stephen Higgs, Manuel V. Borca

**Affiliations:** 1 Agricultural Research Service, U.S. Department of Agriculture, Plum Island Animal Disease Center, Greenport, NY 11944, USA; Jolene.Carlson@ars.usda.gov (J.C.); Vivian.odonnell@ars.usda.gov (V.O.); Marialexia.Alfano@ars.usda.gov (M.A.); Lauro.Velazquez@ars.usda.gov (L.V.S.); Lauren.Holinka@ars.usda.gov (L.G.H.); Peter.Krug@ars.usda.gov (P.W.K.); Douglas.Gladue@ars.usda.gov (D.P.G.); 2 Biosecurity Research Institute and Department of Diagnostic Medicine and Pathobiology, College of Veterinary Medicine, Kansas State University, Manhattan, KS 66506, USA; shiggs@bri.ksu.edu; 3 Department of Pathobiology, University of Connecticut, Storrs, CT 06269, USA

**Keywords:** ASF, ASFV, immune response

## Abstract

African swine fever (ASF) is a lethal hemorrhagic disease of swine caused by a double-stranded DNA virus, ASF virus (ASFV). There is no vaccine to prevent the disease and current control measures are limited to culling and restricting animal movement. Swine infected with attenuated strains are protected against challenge with a homologous virulent virus, but there is limited knowledge of the host immune mechanisms generating that protection. Swine infected with Pretoriuskop/96/4 (Pret4) virus develop a fatal severe disease, while a derivative strain lacking virulence-associated gene *9GL* (Pret4Δ9GL virus) is completely attenuated. Swine infected with Pret4Δ9GL virus and challenged with the virulent parental virus at 7, 10, 14, 21, and 28 days post infection (dpi) showed a progressive acquisition of protection (from 40% at 7 dpi to 80% at 21 and 28 dpi). This animal model was used to associate the presence of host immune response (ASFV-specific antibody and interferon (IFN)-γ responses, or specific cytokine profiles) and protection against challenge. With the exception of ASFV-specific antibodies in survivors challenged at 21 and 28 dpi, no association between the parameters assessed and protection could be established. These results, encompassing data from 65 immunized swine, underscore the complexity of the system under study, suggesting that protection relies on the concurrence of different host immune mechanisms.

## 1. Introduction

African swine fever (ASF) is a contagious viral disease of swine. The causative agent, ASF virus (ASFV), is a large enveloped virus containing a double-stranded (ds) DNA genome of approximately 190,000 base pairs [1,2]. ASFV infections in domestic pigs are often fatal and are characterized by high fever, hemorrhages, ataxia and severe depression [1,2]. Currently, the disease is endemic in sub-Saharan Africa. In Europe, ASF is endemic on the island of Sardinia (Italy), and outbreaks of ASF have been recorded in the Caucasus region since 2007, spreading to neighboring and Eastern European countries [3]. 

There is no vaccine available against ASF, and the only methods of control of ASF are strict quarantine and biosecurity measures, control of animal movement, and slaughter of affected and exposed herds. Experimental vaccines based on the use of an inactivated virus or virus subunits have failed to induce solid protective immunity [3,4,5,6]. Homologous protective immunity does develop in swine surviving infections with moderately virulent or attenuated ASFV isolates [7,8,9,10]. Swine immunized with live attenuated ASFVs containing genetically engineered deletions of specific virulence-associated genes are similarly protected when challenged with homologous parental viruses. Specifically, individual deletions of *UK* (open reading frame (ORF) DP69R), *23-NL* (ORF DP71L), *TK* (ORF A240L) or *9GL* (ORF B119L) genes from the genomes of virulent ASFVs resulted in significant attenuation of these isolates in swine. Animals immunized with these modified viruses demonstrated protection when challenged with homologous ASFV isolates [11,12,13,14]. So far, these observations are the only experimental evidence supporting a rational development of effective live attenuated virus against ASFV.

The deletion of *9GL* (ORF B119L) in the highly virulent ASFV isolates Malawi Lil-20/1, Pretoriuskop/96/4 (Pret4), and more recently Georgia 2007 resulted in complete attenuation of these viruses in swine [11,15,16]. Therefore, targeting the highly conserved *9GL* (ORF B119L) gene for genetic modifications appeared to be a reasonable approach for developing attenuated viruses for use as vaccine candidates. Animals infected with Pret4, lacking the *9GL* gene PretΔ9GL, are all protected when challenged with Pret4 at 42 days post infection (dpi) [15]. In this report, we showed that challenging Pret4Δ9GL infected animals with the parental virulent Pret4 virus earlier showed a progressive acquisition of protective status starting with 40% of the challenged animals at 7 dpi and reaching around 80% of protection between 21 and 28 dpi. Based on these results, we developed an animal model attempting to correlate the presence of host immune response and protection against the challenge. The presence of anti-ASFV antibodies, an ASFV-specific interferon (IFN)-γ response, and circulating cytokines were assessed at different times post Pret4Δ9GL-infection. Results indicate that only the presence of ASFV-specific antibodies correlates with protection in groups challenged at the latest times post infection. These results suggest a complex scenario where protection against disease or infection must result from the presence and interaction of different host immune mechanisms.

## 2. Materials and Methods

### 2.1. Cell Cultures and Viruses

Primary swine macrophage cultures were prepared from defibrinated swine blood as previously described [14]. Briefly, heparin-treated swine blood (50 mL per liter of blood) was incubated at 37 °C for 1 h to allow sedimentation of the erythrocyte fraction. Mononuclear leukocytes were separated by flotation over a Ficoll-Paque-PLUS (GE Healthcare Bio-Sciences, Piscataway, NJ, USA) density gradient (specific gravity 1.077). The monocyte/macrophage cell fraction was cultured in plastic Primaria (Falcon; Corning, Franklin Lakes, NJ, USA) tissue culture flasks containing macrophage media composed of RPMI 1640 (Life Technologies, Grand Island, NY, USA) supplemented with 30% L929 supernatant and 20% fetal bovine serum (HI-FBS, Thermo Scientific, Waltham, MA, USA) for 24 h at 37 °C under 5% CO_2_. Adherent cells were detached from the plastic by using 10 mM ethylenediaminetetraacetic acid (EDTA) in phosphate-buffered saline (PBS) and were then reseeded into Primaria 6- or 96-well plates at a density of 5 × 10^6^ cells per mL for use in assays 24 h later.

Pretoriuskop/96/4 (Pret4) and its derivative harboring a deletion of the *9GL* gene (ORF B119L) Pret4Δ9GL [15] were kindly provided by Dr. John Neilan, from the Plum Island Animal Disease Center, DHS, New York. Pret4Δ9GL was constructed by genetic modification of virulent ASFV Pret4 isolate. A 173-bp region, encompassing amino acid residues 11 to 68 within the *9GL* (ORF B119L) gene was deleted from Pret4 and replaced with a gene cassette containing the β-glucuronidase (GUS) gene under the control of the ASFV p72 late gene promoter (p72-βGUS) by homologous recombination [15].

Comparative growth curves between Pret4 and Pret4Δ9GL viruses were performed in primary swine macrophage cell cultures. Preformed monolayers were prepared in 6-well plates and infected at a multiplicity of infection (MOI) of 0.01 (based on hemadsorption (HAD) 50 titers previously determined in primary swine macrophage cell cultures). After 1 h of adsorption at 37 °C under 5% CO_2_, the inoculum was removed, and the cells were rinsed two times with PBS. The monolayers were then rinsed with macrophage media and incubated for 0, 24, 48, 72 and 96 h at 37 °C under 5% CO_2_. At appropriate times post infection, the cells were frozen at −70 °C, and the thawed lysates were used to determine titers by HAD_50_/mL in primary swine macrophage cell cultures. All samples were run simultaneously to avoid inter-assay variability.

Virus titration was performed on primary swine macrophage cell cultures in 96-well plates. Virus dilutions and cultures were performed using macrophage media. The presence of virus was assessed by HAD, and virus titers were calculated as previously described [17].

### 2.2. Polymerase Chain Reaction 

Identification of virus in the blood of infected/challenged animals was performed by a differential polymerase chain reaction (PCR) based on the recognition of the *9GL* and the *β-Gus* gene. Detection of a *9GL* 160 bp gene fragment was performed using the following pair of primers: forward 5′GTAAGATACGAAAAGGCGTG3′; reverse 5′CATTGGGGACCTAAATAC3′. Detection of a β-Gus 485 bp gene fragment was performed using the following pair of primers: forward 5′GCAATTGCTGTGCCAGGCAGTT3′; reverse 5′TGCCAGTCAACAGACGCGTG3′.

### 2.3. Next Generation Sequencing of ASFV Genomes

ASFV DNA was extracted from infected cells and quantified as described earlier [18]. The full-length sequencing of the virus genome was performed as described [18]. Briefly, one microgram of virus DNA was enzymatically sheared, and the resulting fragmented DNA size distribution was assessed. Adapters and library barcodes were ligated to the fragmented DNA. The appropriate size range of adapter-ligated library was collected using a Pippin Prep™ system (Sage Science, Beverly, MA) followed by normalization of library concentration. The DNA library was then clonally amplified onto Ion Sphere™ Particles (ISPs) and enriched. Enriched template ISPs were prepared for sequencing and loaded onto Ion chips and sequenced with an Ion Personal Genome Machine (PGM) sequencer (Life Technologies, Grand Island, NY, USA). Sequence analysis was performed using Galaxy (https://usegalaxy.org/) and CLC Genomics Workbench (CLCBio).

### 2.4. Detection of Anti-ASFV Antibodies

Anti-ASFV antibodies in sera of infected animals were quantified using two in-house developed assays. The first is a peroxidase assay where Vero cells were infected (MOI = 0.1) with ASFV Vero-adapted Pret4 strain in 96-well plates. Infected cells were fixed in 50% acetone and 50% methanol for 10 min. Plates were blocked with 5% skim milk (Millipore, Billerica, MA, USA) and 0.05% Tween 20 (Sigma, St Louis, MO, USA) for 1 h at 37 °C. Two-fold dilutions of the sera were incubated for 1 h at 37 °C on the 96-well ASFV Vero-infected cell monolayer. After washing with PBS 1×, the presence of anti-ASFV antibodies was detected by using a commercial biotinylated anti-swine IgG, VECTASTAIN Avidin Biotinylated Enzyme Complex, and Vector VIP horseradish peroxidase (Vector; Vector Laboratories, CA, USA). Titers were expressed as the inverse log_10_ of the highest serum dilution reacting with the infected cells.

The second methodology was an indirect enzyme-linked immunosorbent assay (ELISA). Antigen preparation and ELISA procedure were carried out as was previously described [19] with minor adjustments. Briefly, Vero cells were infected with an ASFV adapted to replicate in Vero cells [18] until cytopathic effect reached 100%. The infected cells were re-suspended in water containing protease inhibitor (Roche, New York, NY, USA), followed by the addition of Tween 80 (G-Biosciences, St Louis, MO, USA) and sodium deoxycholate (Sigma, St Louis, MO, USA) to a final concentration of 1% (*v*/*v*). Uninfected Vero cells were treated in the same manner, and these antigens were stored at −70 °C. Maxisorb ELISA plates (Nunc, St Louis, MO, USA) were coated with 1 μg per well of either infected cell or uninfected cell antigen. The plates were blocked with phosphate-buffered saline containing 10% skim milk (Merck, Kenilworth, NJ, USA) and 5% normal goat serum (Sigma). Each swine serum was tested at multiple dilutions against both infected and uninfected cell antigen. ASFV-specific antibodies in the swine sera were detected by an anti-swine IgG-horseradish peroxidase conjugate (KPL, Gaithersburg, MD, USA) and SureBlue Reserve peroxidase substrate (KPL). Plates were read at OD630 nm in an ELx808 plate reader (BioTek, Shoreline, WA, USA). Swine sera were considered positive for ASFV-specific antibodies if the OD630 ratio of the reaction against infected cell antigen to uninfected cell antigen was higher than 2.2.

### 2.5. Detection of ASFV-Specific IFN-γ Producing Cells

Detection of ASFV-specific IFN-γ producing cells was performed using a modification of the ELISpot porcine IFN-γ method (R&D, Minneapolis, MN, USA). Peripheral blood mononuclear cells (PBMCs) were isolated from 15 mL of porcine blood by Ficoll-Paque-PLUS gradient (density 1.077) and centrifugation at 420× *g* for 30 min and washed twice with 1× PBS at room temperature. Cell counts were adjusted to 5 × 10^6^ cells/mL and 100 μL were seeded into 96-well plates. After seeding, cells were stimulated with a buffer containing 25 ng/mL of phorbol 12-myristate 13-acetate (PMA) and 25 ng/mL of calcium ionomycin or were exposed to Vero-adapted-Pret4 virus at MOI = 0.5. The cells were then transferred to ELISpot plates (as provided in the kit) and incubated for 18 h at 37 °C. Steps for washing as well as for using the detection antibody, streptavidin-AP, and BCIP/NBT chromogen were sequentially performed as recommended by the kit’s manufacturer. Spot counts were performed with an ELISpot plate reader (Immunospot, Cellular Technology Limited, Shaker, Heights, OH) with the following settings: counting mask size 100%, normalize counts of mask: Off, sensitivity: 130, min. spot size: 0.086 mm^2^ max. spot size: 0.2596 mm^2^, oversized spots were estimated at spot separation: 1, diffuseness: large, background balance: 67. Cell counts were expressed as a number of spots per 5 × 10^5^ PBMC.

### 2.6. Detection of Cytokines in Sera of Pret4Δ9GL Infected Animals

Levels of serum monocytes chemoattractant protein 2 (MCP2), transforming growth factor beta 1 (TGF-β1), IFN-α, IFN-β, IFN-γ, interleukin 1-α (IL1-α), IL1-β, IL-2, IL-5, IL-8, IL-10, IL-12 p35, IL-12 p40, 2′-5′-oligoadenylate synthetase 1 (OAS), protein kinase R (PKR), tumor necrosis factor (TNF), MX-1, and vascular cell adhesion molecule 1 (VCAM-1) were assessed using commercial ELISAs following manufacturer protocols (MyBioSource, San Diego, CA, USA). Detection of serum cytokines was performed at the time of challenge.

### 2.7. Animal Experiments

Swine experiments were performed under biosafety level 3 conditions in the animal facilities at Plum Island Animal Disease Center (PIADC) following a protocol approved by the Institutional Animal Care and Use Committee. To assess virus replication in vivo, 80 to 90-pound pigs were inoculated intramuscularly (IM) with 10^4^ HAD_50_ with either Pret4Δ9GL or parental Pret4. In addition, Pret4Δ9GL was assessed for its protective effect using 80- to 90-pound commercial breed swine. Groups of pigs were inoculated IM with 10^4^ HAD_50_ of Pret4Δ9GL. At 7, 10, 14, 21 or 28 dpi, animals were challenged IM with 10^4^ HAD_50_ of highly virulent parental Pret4. Clinical signs (anorexia, depression, fever, cyanosis, staggering gait, diarrhea, melena, dyspnea, and cough) and changes in body temperature were recorded daily throughout the 21-day experimental observation period, as previously described [20]. Blood and nasal swabs were collected for virus titrations.

## 3. Results

### 3.1. Analysis of the ASFV Pret4Δ9GL Genome Sequence and Its Parental Pret4 Genome Sequence

To evaluate the accuracy of the genetic modification and the integrity of the genome of the recombinant virus, full genome sequences of Pret4Δ9GL and parental Pret4 viruses were obtained using next generation sequencing (NGS) on the Ion Torrent PGM™ and compared. In summary, Pret4Δ9GL virus did not accumulate any significant mutations during the process of homologous recombination and sequential plaque purification steps. No gene deletion, nor frameshift mutation affecting any viral gene was detected when full-length genomic sequence of Pret4Δ9GL and parental Pret4 viruses were compared (data not shown).

### 3.2. Replication of Pret4 and Pret4Δ9GL In Vitro and In Vivo

**Figure 1:**
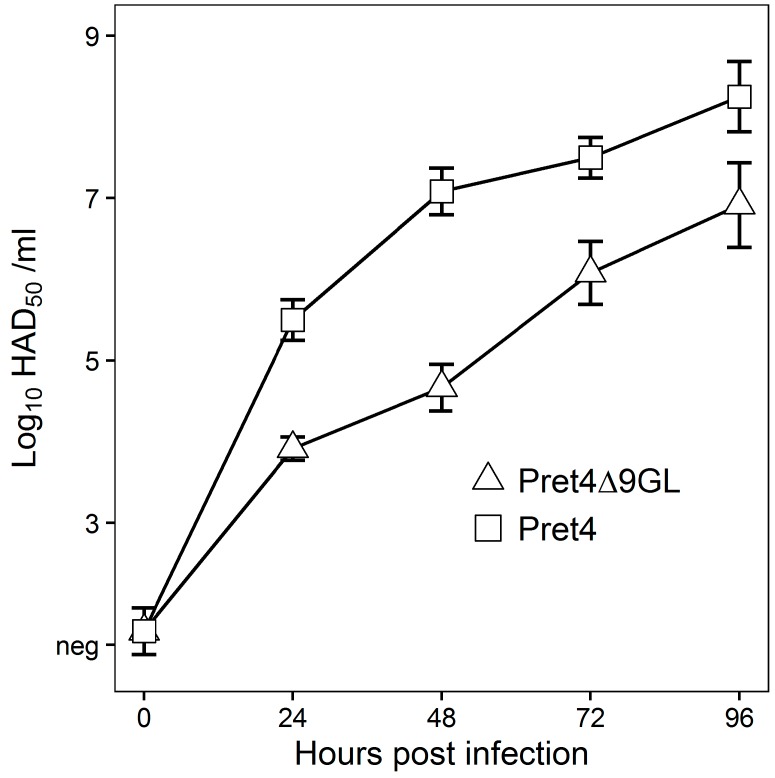
In vitro growth characteristics of Pret4Δ9GL (triangles) and parental Pret4 (squares) viruses. Primary swine macrophage cell cultures were infected (multiplicity of infection (MOI) 0.01) with either Pret4Δ9GL or Pret4 and virus yield titrated at different times post infection. Data represent mean and standard deviation (SD) from three independent experiments. Limit of detection: 1.5 hemadsorption (HAD)_50_/mL. Neg: negative.

In vitro growth characteristics of Pret4Δ9GL virus were evaluated in primary swine macrophage cell cultures, the primary cell targeted by ASFV during infection in swine, and were compared to parental Pret4 in a multistep growth curve (Figure 1). Cell cultures were infected at an MOI of 0.01 and samples were collected at 0, 24, 48, 72 and 96 h post infection (hpi). Pret4Δ9GL growth was significantly delayed compared to the parental Pret4 virus in early time points. Depending on the time point, recombinant virus exhibited titers 10 to 1000-fold lower relative to the parental virus. Therefore, deletion of the 9GL gene significantly affects the ability of the Pret4Δ9GL virus to replicate in vitro in primary swine macrophage cell cultures.

**Figure 2:**
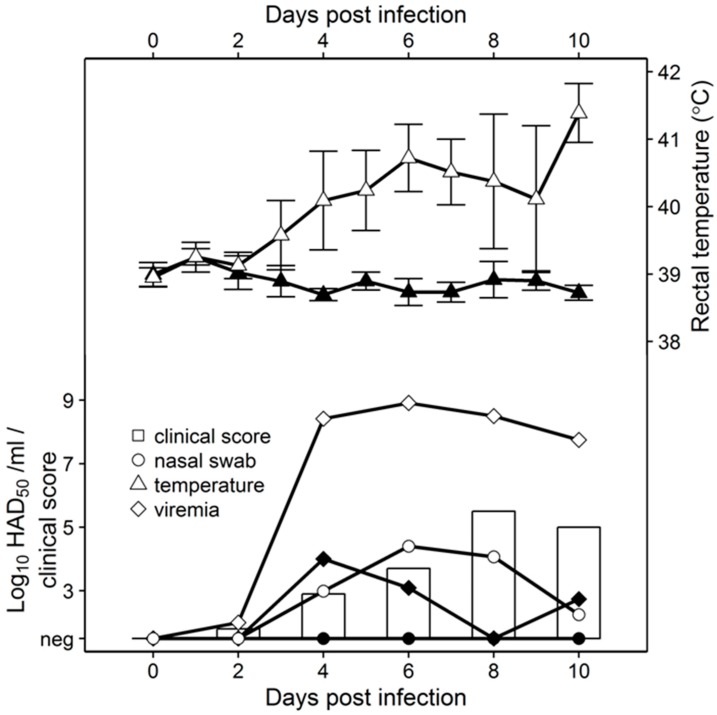
Comparative virulence studies between of Pret4Δ9GL (black symbols) and Pret4 (white symbols and bars). Whole blood and nasal swabs were titrated on primary swine macrophage cell cultures. Limit of detection: 1.5 HAD_50_/mL.

To assess virus replication in vivo, 80- to 90-pound pigs were inoculated IM with 10^4^ HAD_50_ with either Pret4Δ9GL or parental Pret4. Pret4-infected animals exhibited increased body temperature (>40 °C) by 3 to 4 dpi followed by the appearance of clinical signs associated with ASF that progressed over time, and animals either died or were euthanized in extremis 6 to 10 dpi. Swine inoculated with Pret4Δ9GL remained clinically normal during the observational period (Figure 2).

As clinical disease progressed, viremia and viral shedding in Pret4-infected animals began around 4 dpi and remained high until death. Viremia in animals inoculated with Pret4Δ9GL peaked transiently by 4 dpi and remained at significantly lower levels than in Pret4-infected animals. No nasal viral shedding was detected in Pret4Δ9GL-infected animals.

### 3.3. Onset of Protective Immunity in Animals Infected with Pret4 and PretΔ9GL

**Figure 3:**
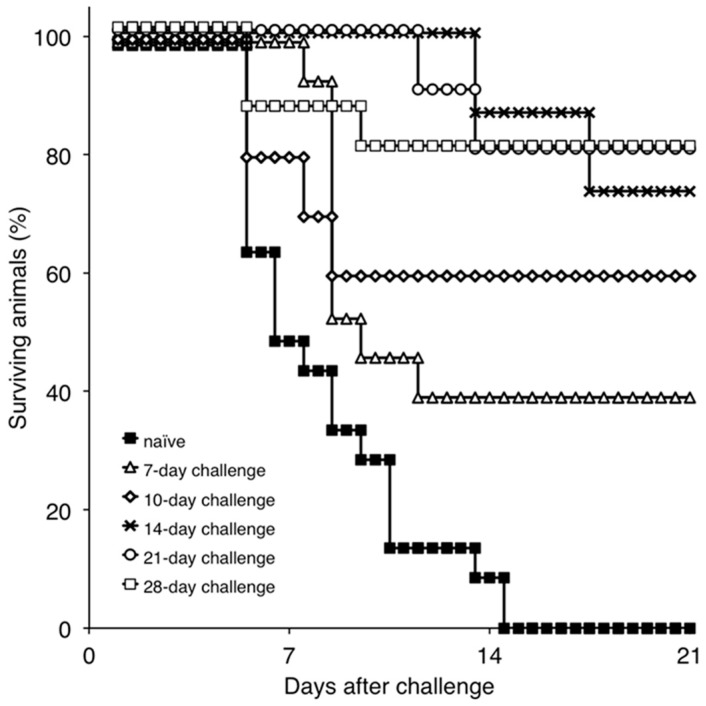
Percentage of surviving animals challenged with Pret4 virus at different times post intramuscular (IM) inoculation with Pret4Δ9GL. Swine were infected with 10^4^ HAD_50_/mL Pret4Δ9GL virus and challenged with virulent Pret4 (same dose and route) at either 7 (*n* = 15), 10 (*n* = 10), 14 (*n* = 15), 21 (*n* = 10), or 28 (*n* = 15) days later.

To gain an understanding of immune mechanisms present in ASFV protected animals, we attempted to develop a model that would allow us to examine the correlation between the presence of immunological parameters and protection against the challenge. Therefore, the onset of protection in Pret4Δ9GL-infected animals was assessed for the purpose of obtaining experimental groups where protected and unprotected animals coincided. Consequently, groups of swine (*n* = 4–6, depending on the group, experiments were repeated two or three times) were infected IM with Pret4Δ9GL virus at 10^4^ HAD_50_ and challenged with the same dose and route with virulent parental Pret4 virus at different times post Pret4Δ9GL virus infection. Results demonstrated that, surprisingly, six out 15 (40%) of the animals challenged as early as seven days post Pret4Δ9GL virus infection survived the challenge. When swine inoculated with Pret4Δ9GL virus were challenged at later time points, we observed an increase in the proportion of animals protected against the challenge. As a result, six out of 10 (60%), 11 out 15 (73.33%), eight out 10 (80%), and 12 out 15 (80%) swine survived when challenged at 10, 14, 21, and 28 dpi with Pret4Δ9GL (Figure 3).

**Figure 4:**
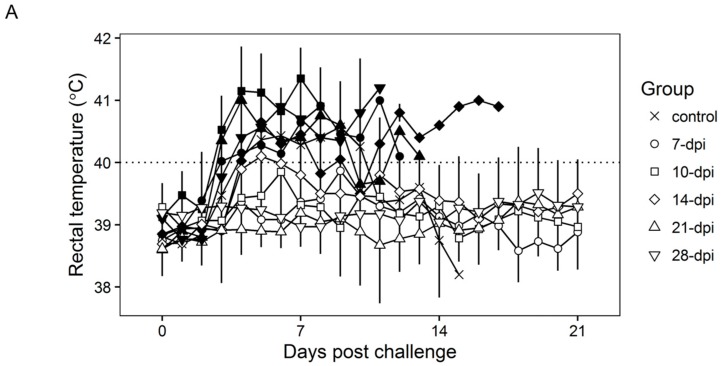

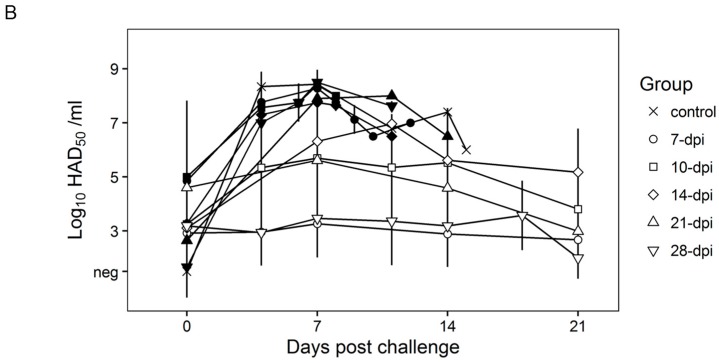
Rectal temperature (**A**) and viremia (**B**) in Pret4Δ9GL virus-infected animals challenged with Pret4 virus at different times. Average and SD of each group along a 21-day observational period is presented. Viremia values are expressed as log_10_ HAD_50_/mL. Limit of detection: 1.5 HAD_50_/mL. Survival status of swine is indicated as survived (white open shapes) or did not survive (black filled shapes). Dpi: days post infection.

Surviving swine, regardless of the time of the challenge, all present a relatively good health showing only very transient raises of body temperature (1–6 days) without any severe clinical signs associated with the disease (Figure 4A). Most swine succumbing to the challenge present similar kinetic patterns of clinical signs which closely resemble those of the challenged naïve animals, starting by day 3–5 post challenge (pc) and humanely ending by day 8–10 pc (Figure 3 and Figure 4A). A few swine in the 14, 21, and 28-day challenge groups had a delayed onset of clinical disease and were humanely euthanized at 11–18 days post challenge. Consistent with the presentation of clinical signs associated with the disease, the kinetics of viremia in all succumbing animals practically overlaps those of challenged naïve animals peaking around day 7 pc and remaining high until animals were euthanized (Figure 4B). Viremia in animals surviving the challenge is present with a broad range of titers (10^3^ to 10^8^ log_10_ HAD_50_/mL peaking at day 7–10 pc) in all groups at almost all the time points tested during the observational period of 21 days pc (Figure 4B).

Therefore, it appears that clinical presentation of the disease, as well as the kinetics of virus replication in non-protected swine, are very similar regardless of the time of challenge. This is somewhat surprising since it might be expected that immune mechanisms developed at different times post Pret4Δ9GL virus infection could be quantitatively or qualitatively different. Therefore, it could be expected that the level or type of protection achieved could be different, and that difference may be reflected in the outcome of disease/viremia produced in either protected or non-protected animals challenged at different times post Pret4Δ9GL virus infection.

To understand the basis behind protection against the challenge present in each of the time points after the Pret4Δ9GL virus infection we attempted to correlate different virological and immunological parameters with the clinical outcome observed after the challenge. We particularly focused on quantifying at the time of challenge the following parameters: Pret4Δ9GL viremia, circulating anti-ASFV antibodies, circulating ASFV-specific IFN-γ producing cells, and levels of several cytokines and chemokines, which play a role in the innate immune response.

### 3.4. Presence and Levels of Pret4Δ9GL Viremia at the Moment of Challenge

**Figure 5:**
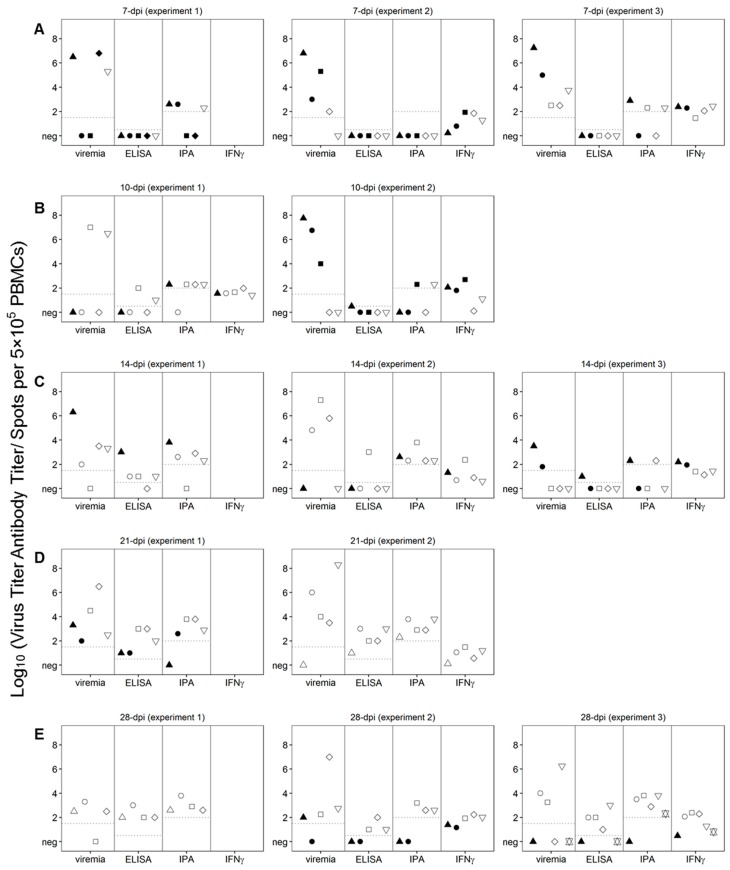
Viremia and immunological parameters at the time of challenge, day zero, in animals infected with Pret4Δ9GL. Values of viremia, anti-African swine fever virus (ASFV) antibody titers detected by ELISA and immunoperoxidase (IPA), and the number of circulating ASFV-specific interferon (IFN)-γ-producing cells are presented for each individual pig. Survival status of swine is indicated as survived (white open shapes) or did not survive (black filled shapes). Pret4Δ9GL virus infected-animals were challenged at 7 (**A**), 10 (**B**), 14 (**C**), 21 (**D**), or 28 (**E**) days later. PBMCs: peripheral blood mononuclear cells.

It is possible that infection with Pret4Δ9GL as a competitive factor may play a role in the process of protection against the challenge with virulent Pret4 virus. Therefore, the correlation between the presence of Pret4Δ9GL viremia at the time of challenge in protected swine was analyzed in all of the groups. Pret4Δ9GL viremia titers at the time of challenge are quite heterogeneous (ranging from undetectable to around 7 log_10_ HAD_50_/mL) in all of the groups considered (animals challenged at 7, 10, 14, 21 and 28 days post Pret4Δ9GL virus infection). This heterogeneity in viremia is found in all swine independent of their survival status after challenge (Figure 5A–E). Of particular importance is that in each of the groups, on average, animals surviving the challenge have viremia titers ranging from as low as negative (≤1.5 log_10_ HAD_50_/mL) to values as high as 4–7 log_10_ HAD_50_/mL. Therefore, no clear correlation could be established between the presence of Pret4Δ9GL viremia at the time of challenge and protection against virulent challenge.

### 3.5. Presence and Levels of Anti-ASFV Antibodies at Day 0, the Time of Challenge

The antibody response against ASFV antigens was monitored at the time of challenge of the Pret4Δ9GL-infected animals. ASFV-specific antibody response was detected using an indirect ELISA and an immunoperoxidase assay (IPA). In all 15 animals challenged at 7 dpi with Pret4Δ9GL, antibodies were undetectable by ELISA, regardless of survival status. In nine of the animals in the 7-day challenge group, antibodies were still undetectable by IPA, although three of the nine were protected against the challenge. Of the six surviving animals in this group, three had similar antibody titers by IPA compared to three animals that were not, protected (Figure 5A). Out of the 10 animals challenged at 10 dpi with Pret4 virus, seven of them were still negative by ELISA, with six of them protected against the challenge. Only two protected animals challenged at 10 days demonstrated a positive result by ELISA. Two of the non-protected animals showed positive IPA titers as well as four of the six protected ones (Figure 5B). Similarly, three of the four animals not protected when challenged at 14 days post Pret4Δ9GL had antibody detected by IPA, and two out of four had antibody detected by ELISA. Of the protected swine, four out of 11 animals had measurable antibody by ELISA, and eight out of 11 swine had measurable antibody by IPA, suggesting IPA is more sensitive than ELISA (Figure 5C). Therefore, in animals challenged at days 7, 10 and 14 days post Pret4Δ9GL infection, the presence of antibody does not correlate with protection against the virulent challenge. Analysis of ASFV-specific antibodies in animals challenged at later times is more closely correlated with protection against the virulent challenge. Antibody titers in non-protected animals challenged at 21 days post Pret4Δ9GL infection are lower or absent when detected either by ELISA or IPA (Figure 5D). Evidently, in the group challenged at 28 days post Pret4Δ9GL infection, all protected animals presented antibody titers both by ELISA and IPA, while the only three unprotected swine did not have detectable anti-ASFV antibodies by either methodology (Figure 5E).

### 3.6. Presence and Levels of ASFV-Specific IFN-γ at the Time of Challenge

As a marker of T-cell sensitization the ASFV-specific IFN-γ response at the time of challenge was evaluated in PretΔ9GL-infected animals. PBMCs producing IFN-γ were detected by ELISpot (because of technical limitations not all animals in each of the groups were analyzed). In the 10 animals tested within the group challenged at 7 dpi, the response was present at similar levels in five animals surviving the challenge as well as in the other five that succumbed (Figure 5A). All 10 animals challenged at days 10 post Pret4Δ9GL virus infection were evaluated for IFN-γ response. No significant differences were found in the number of IFN-γ producing cells between the six protected animals and the other four that succumbed to the challenge (with the exception that one of the protected animals did not show any detectable activity) (Figure 5B). Similarly, IFN-γ responses evaluated in 10 animals challenged at 14 days post Pret4Δ9GL virus infection were not markedly different between the three succumbing to the infection and the other seven surviving challenge. (Figure 5C). All five animals tested for the presence of IFN-γ producing cells in the group challenged at 21 days post Pret4Δ9GL infection were protected, with four of them presenting IFN-γ activity (Figure 5D). Finally, in the group challenged at 28 days post inoculation with Pret4Δ9GL where IFN-γ was measured in 11 swine, 10 animals tested showed no significant differences in their IFN-γ activity although eight were protected and three of them did not survive the challenge (Figure 5E). One animal that did not survive in experiment 3 had the lowest levels of IFN-γ. As a result, there is no direct association between the presence of IFN-γ activity and protection against the challenge with virulent virus, particularly when challenge is performed at early times post Pret4Δ9GL infection.

### 3.7. Cytokine Profile at Day 0, the Time of Challenge, in Serum of Animals Infected with Pret4Δ9GL

**Figure 6:**
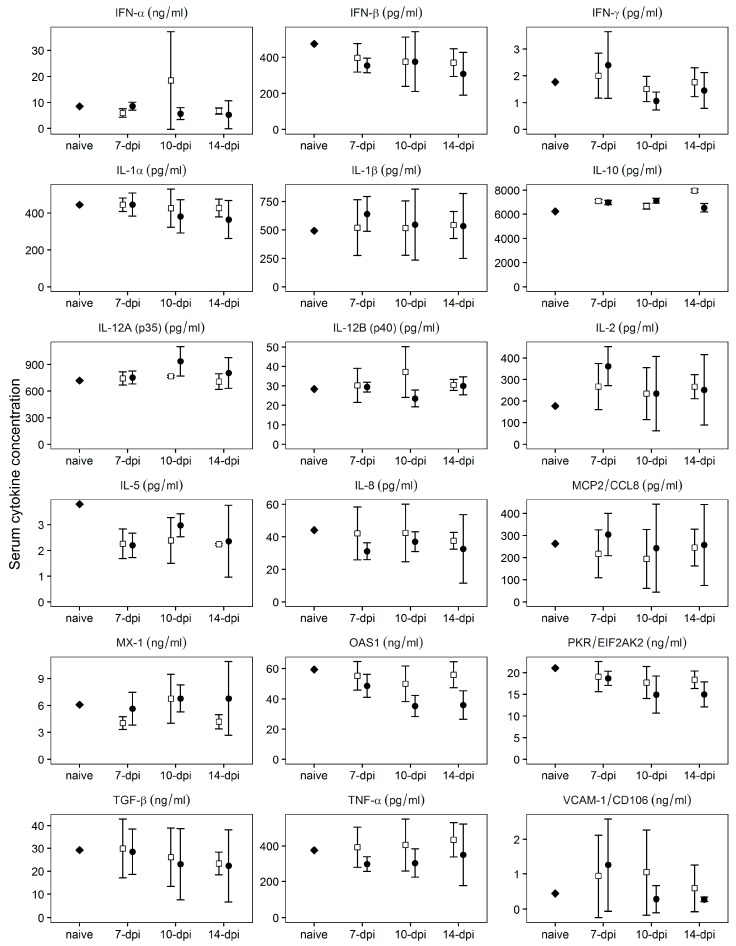
Evaluation of the systemic levels of different host cytokines at the time of challenge in swine infected with Pret4Δ9GL virus. Average and 95% confidence intervals of 15, 10, and 15 animals from groups challenged at 7, 10, and 14 days post Pret4Δ9GL infection are presented. Values are expressed as concentration per mL of serum determined as described in material and methods. IFN, interferon; IL, interleukin; MCP2, monocytes chemoattractant protein 2; OAS1, 5′-oligoadenylate synthetase 1; PKR, protein kinase R; TGF, transforming growth factor; TNF, tumor necrosis factor; VCAM-1, vascular cell adhesion molecule 1.

Protection against the challenge with virulent Pret4Δ9GL at early times (7–14 days) post infection with Pret4Δ9GL has not been previously described in animals infected with attenuated ASFV strains. Therefore, we tried to establish a correlation between specific patterns of innate host immune response mediators and protection against the challenge in Pret4Δ9GL virus-infected animals. Levels of MCP2, TGF-β1, IFN-α, IFN-β, IFN-γ, IL1-α IL1-β, IL-2, IL-5, IL-6, IL-8, IL-10, IL-12 p35, IL-12 p40, OAS, PKR, TNF-α, MX-1, and VCAM were assessed using commercial ELISAs following manufacturer protocols in sera of animals challenged at early times (7, 10 and 14 days) post Pret4Δ9GL virus infection. Generally, all cytokine circulating levels tested were quite heterogeneous in every group considered (Figure 6).

Regardless of heterogeneity, the average values do not vary significantly (*p* ≥ 0.05) between protected and non-protected animals for any of the cytokine assessed, and in general, those values do not significantly (*p* ≥ 0.05) differ from those of naïve animals. Therefore, no positive correlation could be established between the level of any of the measured cytokines and protection of the Pret4Δ9GL virus infected animals at the time of challenge with the parental virulent ASFV Pret4.

## 4. Discussion

Successful experimental ASFV vaccines have been exclusively based on the use of attenuated virus strains, regardless of the origin of attenuation i.e., natural, acquired by serial passages in alternative hosts or by genetic manipulation [5,7,10,11,15,21,22,23]. In all these reports, the challenge was performed no sooner than three weeks after vaccination. Importantly, the host immune mechanisms mediating protection in animals infected with attenuated strains are not characterized. In fact, there are very few reports investigating this critical issue. The only direct evidence about mechanisms of protection against ASFV is derived from reports where the acquisition of protective humoral immunity by naïve pigs against ASFV was achieved after passive transfer of ASFV-specific antibodies obtained from pig donors that were previously infected with attenuated ASFV [10,24]. As it relates to cellular immunity, the only direct evidence of mechanisms of protection is derived from a report demonstrating that partial CD8+ T-cell depletion impedes the development of a host protective immune response in challenged animals that have been previously vaccinated with attenuated ASFV [25]. The presence of circulating virus-neutralizing antibodies in animals infected or vaccinated with attenuated ASFV strains has been a controversial issue [10,15,24,26,27,28]. Moreover, there have been contradictory reports regarding the efficacy of neutralizing antibodies in preventing infection of disease [15,26]. Lately, a possible correlation between the induction of virus-specific circulating IFN-γ-producing cells and protection in animals vaccinated with attenuated ASFV strains has been reported [21,29]. Therefore, there is no agreement on the host immune mechanisms mediating protection in animals vaccinated with attenuated strains.

In this report, we present data based on the use of an experimental model consisting of 65 swine (20 naïve controls) vaccinated with a rationally attenuated virus strain where we analyze the presence of different host immunological parameters and try to correlate them with protection against the challenge with a virulent homologous strain at different times post vaccination. This model allowed us to analyze, at different times post vaccination, the immunological status of animals that did or did not survive the challenge. The purpose of performing challenges at different times post infection with the attenuated strain, is to allow a monitoring of the presence of different immune parameters that may change during the maturation of the host response between day 0 and 28 post Pret4Δ9GL virus infection. In fact, it was unexpected that 40% and 60% of animals challenged as early as 7 and 10 days post Pret4Δ9GL virus infection survived. The presence of such early protection in animals infected with an attenuated strain of ASFV was never described before and clearly indicates the existence of at least partially effective early host immune mechanisms. Unfortunately, none of the host parameters analyzed in this report within the first two weeks after Pret4Δ9GL virus infection showed a direct correlation with protection against challenge.

It is expected that immune mechanisms may suffer quantitative and qualitative changes along the time post Pret4Δ9GL virus infection. In fact, there is an acquisition of better protective immunity that is reflected in an increased percentage of protection at later times post Pret4Δ9GL virus infection. It is clear that at 21–28 days post Pret4Δ9GL virus inoculation, ASFV-specific antibody levels became a reliable indicator of protection.

The presence of virus-specific circulating IFN-γ-producing cells is not a reliable predictor of protection since no direct correlation could be established between the number of IFN-γ-producing cells and protection at any of the different times post Pret4Δ9GL virus infection evaluated. This is in contradiction with other reports [21,29] stating a direct correlation between circulating IFN-γ producing cells and protection against challenge in animals. At this moment, the methodological basis producing these contradictory results is not clear for us. Similarly, no correlation could be established between any particular sera cytokine patterns in any of the groups with protection. Rather, the heterogeneity in the cytokine levels observed in all groups, regardless of the time after Pret4Δ9GL or the survival status of each group of animals, is actually surprising.

**Figure 7:**
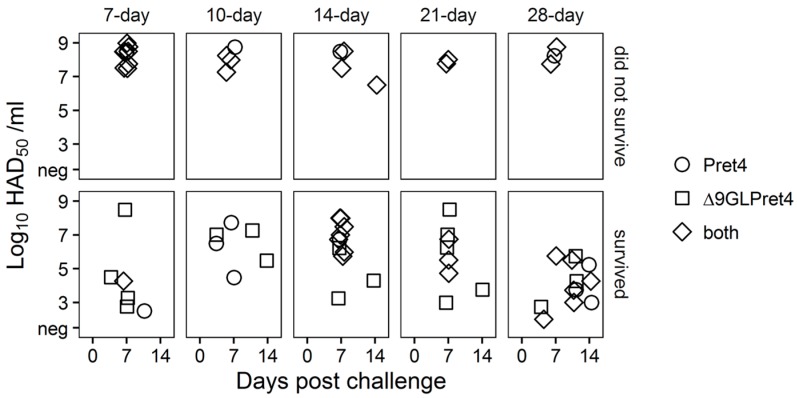
Characterization of circulating virus after the challenge in animals infected with Pret4Δ9GL. Identification of the presence of Pret4Δ9GL and Pret4 virus was performed by differential PCR based on the detection of β-Gus and 9GL genes as markers for Pret4Δ9GL and Pret4 virus. The determination was performed using the blood sample with the highest viremia value, after challenge, found in that animal.

It is interesting that in most of the cases the absence of ASFV-related clinical signs does not imply protection against the systemic replication of the challenge virus. Approximately, half of the animals surviving the challenge presents Pret4 virus in blood, by itself or accompanied by the presence of Pret4Δ9GL virus (Figure 7).

Therefore, systemic distribution of the challenge virus does not necessary imply the development of the disease. We already observed this in animals infected with attenuated Georgia 2007 variants. Presence and level of viremia induced by the challenge virus do not correlate with the severity of the disease [16,23].

In summary, we have attempted to correlate different immunological parameters with protection against challenge in animals infected with an attenuated strain of ASFV. Although the presence of virus-specific antibodies correlates with protection when challenge is performed three weeks or later post infection with the attenuated strain, at this time we could not clearly identify the host mechanism mediating protection at the early time points. This study, encompassing data from 65 vaccinated swine, highlights a complex scenario where protection against disease or infection must result from the presence and interaction of different host immune mechanisms. Further research evaluating other host parameters will be necessary to clarify the events resulting in early protection. 

## References

[1] Costard S., Wieland B., de Glanville W., Jori F., Rowlands R., Vosloo W., Roger F., Pfeiffer D.U., Dixon L.K. (2009). African swine fever: How can global spread be prevented?. Philos. Trans. R. Soc. Lond. Ser. B Biol. Sci..

[2] Tulman E.R., Delhon G.A., Ku B.K., Rock D.L. (2009). African swine fever virus. Lesser Known Large dsDNA Viruses.

[3] Chapman D.A., Darby A.C., Da Silva M., Upton C., Radford A.D., Dixon L.K. (2011). Genomic analysis of highly virulent Georgia 2007/1 isolate of African swine fever virus. Emerg. Infect. Dis..

[4] Forman A.J., Wardley R.C., Wilkinson P.J. (1982). The immunological response of pigs and guinea pigs to antigens of African swine fever virus. Arch. Virol..

[5] Kihm U.A.M., Mueller H., Pool R. (1987). Approaches to Vaccination African Swine Fever.

[6] Lacasta A., Ballester M., Monteagudo P.L., Rodriguez J.M., Salas M.L., Accensi F., Pina-Pedrero S., Bensaid A., Argilaguet J., Lopez-Soria S. (2014). Expression library immunization can confer protection against lethal challenge with African swine fever virus. J. Virol..

[7] Hamdy F.M., Dardiri A.H. (1984). Clinical and immunologic responses of pigs to African swine fever virus isolated from the western hemisphere. Am. J. Vet. Res..

[8] Lacasta A., Monteagudo P.L., Jimenez-Marin A., Accensi F., Ballester M., Argilaguet J., Galindo-Cardiel I., Segales J., Salas M.L., Dominguez J. (2015). Live attenuated African swine fever viruses as ideal tools to dissect the mechanisms involved in viral pathogenesis and immune protection. Vet. Res..

[9] Mebus C.A. (1988). African swine fever. Adv. Virus Res..

[10] Gonzalvo F.R., Carnero M.E., Bruyel V. (1981). Immunological Responses of Pigs to Partially Attenuated ASF and Their Resistance to Virulent Homologous and Heterologous Viruses.

[11] Lewis T., Zsak L., Burrage T.G., Lu Z., Kutish G.F., Neilan J.G., Rock D.L. (2000). An African swine fever virus ERV1-ALR homologue, 9GL, affects virion maturation and viral growth in macrophages and viral virulence in swine. J. Virol..

[12] Moore D.M., Zsak L., Neilan J.G., Lu Z., Rock D.L. (1998). The African swine fever virus thymidine kinase gene is required for efficient replication in swine macrophages and for virulence in swine. J. Virology.

[13] Zsak L., Caler E., Lu Z., Kutish G.F., Neilan J.G., Rock D.L. (1998). A nonessential African swine fever virus gene *UK* is a significant virulence determinant in domestic swine. J. Virol..

[14] Zsak L., Lu Z., Kutish G.F., Neilan J.G., Rock D.L. (1996). An African swine fever virus virulence-associated gene *NL-S* with similarity to the herpes simplex virus *ICP34.5* gene. J. Virol..

[15] Neilan J.G., Zsak L., Lu Z., Burrage T.G., Kutish G.F., Rock D.L. (2004). Neutralizing antibodies to African swine fever virus proteins p30, p54, and p72 are not sufficient for antibody-mediated protection. Virology.

[16] O’Donnell V., Holinka L.G., Gladue D.P., Sanford B., Krug P.W., Lu X., Arzt J., Reese B., Carrillo C., Risatti G.R. (2015). African swine fever virus georgia isolate harboring deletions of *MGF360* and *MGF505* genes is attenuated in swine and confers protection against challenge with virulent parental virus. J. Virol..

[17] Reed L.J., Muench H. (1938). A simple method of estimating fifty percent endpoints. Am. J. Hyg..

[18] Krug P.W., Holinka L.G., O’Donnell V., Reese B., Sanford B., Fernandez-Sainz I., Gladue D.P., Arzt J., Rodriguez L., Risatti G.R. (2015). The progressive adaptation of a georgian isolate of African swine fever virus to vero cells leads to a gradual attenuation of virulence in swine corresponding to major modifications of the viral genome. J. Virol..

[19] Katz D., Shi W., Wildes M.J., Krug P.W., Hilliard J.K. (2012). Reassessing the detection of b-virus-specific serum antibodies. Comp. Med..

[20] Howey E.B., O’Donnell V., de Carvalho Ferreira H.C., Borca M.V., Arzt J. (2013). Pathogenesis of highly virulent African swine fever virus in domestic pigs exposed via intraoropharyngeal, intranasopharyngeal, and intramuscular inoculation, and by direct contact with infected pigs. Virus Res..

[21] King K., Chapman D., Argilaguet J.M., Fishbourne E., Hutet E., Cariolet R., Hutchings G., Oura C.A., Netherton C.L., Moffat K. (2011). Protection of European domestic pigs from virulent African isolates of african swine fever virus by experimental immunisation. Vaccine.

[22] Leitao A., Cartaxeiro C., Coelho R., Cruz B., Parkhouse R.M., Portugal F., Vigario J.D., Martins C.L. (2001). The non-haemadsorbing African swine fever virus isolate ASFV/NH/P68 provides a model for defining the protective anti-virus immune response. J. Gen. Virol..

[23] O’Donnell V., Holinka L.G., Krug P.W., Gladue D.P., Carlson J., Sanford B., Alfano M., Kramer E., Lu Z., Arzt J. (2015). African swine fever virus Georgia 2007 with a deletion of virulence-associated gene *9GL* (B119L), when administered at low doses, leads to virus attenuation in swine and induces an effective protection against homologous challenge. J. Virol..

[24] Onisk D.V., Borca M.V., Kutish G., Kramer E., Irusta P., Rock D.L. (1994). Passively transferred African swine fever virus antibodies protect swine against lethal infection. Virology.

[25] Oura C.A., Denyer M.S., Takamatsu H., Parkhouse R.M. (2005). In vivo depletion of CD8+ T lymphocytes abrogates protective immunity to African swine fever virus. J. Gen. Virol..

[26] Gomez-Puertas P., Rodriguez F., Oviedo J.M., Brun A., Alonso C., Escribano J.M. (1998). The African swine fever virus proteins p54 and p30 are involved in two distinct steps of virus attachment and both contribute to the antibody-mediated protective immune response. Virology.

[27] Ruiz Gonzalvo F., Caballero C., Martinez J., Carnero M.E. (1986). Neutralization of African swine fever virus by sera from African swine fever-resistant pigs. Am. J. Vet. Res..

[28] Ruiz Gonzalvo F., Carnero M.E., Caballero C., Martinez J. (1986). Inhibition of African swine fever infection in the presence of immune sera in vivo and in vitro. Am. J. Vet. Res..

[29] Argilaguet J.M., Perez-Martin E., Nofrarias M., Gallardo C., Accensi F., Lacasta A., Mora M., Ballester M., Galindo-Cardiel I., Lopez-Soria S. (2012). DNA vaccination partially protects against African swine fever virus lethal challenge in the absence of antibodies. PLoS ONE.

